# Do Differential Response Rates to Patient Surveys Between Organizations Lead to Unfair Performance Comparisons? Evidence From the English Cancer Patient Experience Survey

**DOI:** 10.1097/MLR.0000000000000457

**Published:** 2015-12-18

**Authors:** Catherine L. Saunders, Marc N. Elliott, Georgios Lyratzopoulos, Gary A. Abel

**Affiliations:** * Cambridge Centre for Health Services Research, University of Cambridge; † RAND Europe, Cambridge, UK; ‡ RAND Corporation, Santa Monica, CA; § Department of Epidemiology & Public Health, Health Behaviour Research Centre, University College London, London, UK

**Keywords:** survey, nonresponse, healthcare quality, patient experience, cancer

## Abstract

**Background:**

Patient surveys typically have variable response rates between organizations, leading to concerns that such differences may affect the validity of performance comparisons.

**Objective:**

To explore the size and likely sources of associations between hospital-level survey response rates and patient experience.

**Research Design, Subjects, and Measures:**

Cross-sectional mail survey including 60 patient experience items sent to 101,771 cancer survivors recently treated by 158 English NHS hospitals. Age, sex, race/ethnicity, socioeconomic status, clinical diagnosis, hospital type, and region were available for respondents and nonrespondents.

**Results:**

The overall response rate was 67% (range, 39% to 77% between hospitals). Hospitals with higher response rates had higher scores for all items (Spearman correlation range, 0.03–0.44), particularly questions regarding hospital-level administrative processes, for example, procedure cancellations or medical note availability.

From multivariable analysis, associations between individual patient experience and hospital-level response rates were statistically significant (*P*<0.05) for 53/59 analyzed questions, decreasing to 37/59 after adjusting for case-mix, and 25/59 after further adjusting for hospital-level characteristics.

Predicting responses of nonrespondents, and re-estimating hypothetical hospital scores assuming a 100% response rate, we found that currently low performing hospitals would have attained even lower scores. Overall nationwide attainment would have decreased slightly to that currently observed.

**Conclusions:**

Higher response rate hospitals have more positive experience scores, and this is only partly explained by patient case-mix. High response rates may be a marker of efficient hospital administration, and higher quality that should not, therefore, be adjusted away in public reporting. Although nonresponse may result in slightly overestimating overall national levels of performance, it does not appear to meaningfully bias comparisons of case-mix-adjusted hospital results.

## Abstract 2

Supplemental Digital Content is available in the text.

Patient experience is a critical dimension of high-quality care.^1^,^2^ Consequently, nationwide surveys are increasingly used to measure the experience of large numbers of patients, although complete (100%) response rates are never achievable. Concerns about differential nonresponse between organizations can impede stakeholder engagement with the survey findings,^3^,^4^ weakening the effectiveness of policies (such as public reporting) that aim to incentivize quality improvement. Evaluation of the consequences of nonresponse in patient experience surveys can empirically examine the validity of such concerns.^5^,^6^

Differences in nonresponse between health care organizations might suggest a need to adjust for organization-level response rates in public reporting schemes. Variation in organization response rates may reflect chance, patient case-mix differences, or differences in survey delivery between organizations.^7^,^8^ Alternatively, it may reflect an intrinsic association between patient experience and survey response at the level of individual patients; patients who had a positive experience may be more inclined to respond to surveys,^9^,^10^ or return them more quickly,^6^ or vice versa. Further, such an endogenous relationship may also be present at the organization level, such that organization characteristics or behavior of hospitals promoting better care may also increase response rates, or vice versa.

If, after accounting for differences in patient case-mix (and survey mode when needed), no association between hospital survey response rates and hospital performance measures can be observed, concerns about potential nonresponse bias in organizational performance comparisons are lessened.^8^,^11^,^12^ In other words, if response rates and performance are not correlated at all then it is unlikely that nonresponse is the dominant driver of variation in performance between organisations.^13^ To do this, important case-mix variables must be collected for responders, and specified appropriately, as is typical for patient experience surveys such as GPPS^12^ and HCAHPS surveys.^8^ Typically, measures of age, health status, and socioeconomic status are relevant.

Where a correlation is observed, interpretation is substantially more complex; nonresponse bias may be present. Response rate alone, however, is a problematic indicator of the strength of any possible bias; the most obvious example here being that when nonresponse occurs completely at random then findings will be unbiased even at very low response rates.^7^,^10^

Against this background, this work is presented in the context of the high profile organizational comparisons supported by the English Cancer Patient Experience Survey (CPES).^14^–^16^ CPES has a response rate that is high overall (67%), particularly in comparison with other national hospital-based patient experience surveys from the UK (the Adult Inpatient Survey, response rate 49%)^17^ or the US (HCAHPS, response rate 33%),^18^ however, it is also variable between hospitals. We examine the presence, direction, and size of associations between hospital performance and the hospital survey response rate and consider how much concern this gives about nonresponse bias for hospital performance comparisons from this survey. Using multivariable regression, analyzing survey responses, and information about nonresponders from hospital records, we answer 4 research questions:

1. How much of the variability in hospital survey response rates can be explained by chance alone, or by the case-mix (ie, the sociodemographic and clinical profile) of the patients attending each hospital?
2. What are the hospital-level correlations between hospital patient experience performance scores and hospital survey response rates?
3. What is the association between individuals’ patient experience and hospital survey response rates, after accounting for both patient and hospital characteristics?
4. What would the hypothetical crude patient experience performance score for each hospital be at a 100% response rate, and what would the correlation between patient experience and survey response rate be in this situation?

## METHODS

### Data

CPES is a mail survey of all patients with cancer as the primary recorded diagnosis during an episode of inpatient or outpatient treatment at an English NHS hospital, most similar to a general acute care hospital in the United States, during a 3-month period, and has been fielded annually since 2010.^17^ The survey is commissioned by NHS England and is implemented by a single commercial survey provider, Quality Health (Chesterfield). All patients receive a survey addressed from the hospital at which they received treatment, and initial nonrespondents receive up to 2 mail reminders.^19^ Responses received within 4 months of the initial mailing are included in the final survey analysis sample. Individual patient-level survey data for 2010 are available from the UK Data Archive^20^ and the anonymous dataset with the characteristics of all patients sent the survey for the same year was provided to the study authors by the survey provider.

### Patient Experience Outcome Measures

The 2010 survey contained 60 evaluative questions covering cancer patient experience, from primary care before diagnosis through inpatient and outpatient hospital experience and postdischarge care. Questions were developed by the survey provider and testing carried out by a panel of volunteer cancer patients.^19^ Public reporting of this survey is based on dichotomized positive or negative experience categorizations,^17^ and the same classification is used in this analysis. Hospital performance for each item was defined as the proportion of patients endorsing a positive experience rating at each hospital.

### Response Rate

The survey response rate (overall, and by hospital) is calculated as the proportion of eligible patients (ie, not those who had moved house, died, or were otherwise ineligible) who returned a completed survey (AAPOR response rate 2).^21^

### Sociodemographic and Case-mix Measures, Hospital Characteristics

Hospital records included age (in 10 y age groups), sex, cancer diagnosis based on ICD10 coding (36 groups), and socioeconomic deprivation (5 groups) based on residential neighborhood, the index of multiple deprivation,^22^ for all patients (respondents and nonrespondents). Self-reported ethnic group, following the English Office of National Statistics 2001 six-group classification (White, Mixed, Asian or Asian British, Black or Black British, Chinese, or Other) was available for survey respondents, and ethnicity from hospital records was available for both respondents and nonrespondents. For the 158 NHS hospitals, region and type (Specialist, Teaching and Small, Medium, or Large Acute hospitals) were recorded.

### Survey Response Time

On the basis of the survey provider date system for logging the survey responses, a measure of the length of time taken to return the survey was available for respondents, allowing analyses based on the assumption that late respondents are more similar to nonrespondents than early.^6^ This is described in full in Supplemental Digital Content 1 (http://links.lww.com/MLR/B51).

### Analysis

Four analyses were performed, as described below. Briefly, the 4 analyses attempted to do the following: (1) Quantify how much of the variation in hospital survey response rates could be explained by chance or case-mix. (2) Describe the crude hospital-level correlation between hospital performance scores and hospital response rates. (3) Explore the association between hospital survey response rates and individuals’ patient experience, adjusting for both patient and hospital characteristics. This third analysis differs from the first as it explores patient-level patient experience rather than hospital-level survey response as the outcome. (4) Predict the patient experience of nonrespondents and estimate the hypothetical unadjusted performance of each hospital at a 100% response, a simulation of the second analysis without nonresponse. Details of how each analysis maps onto our research questions and our modeling approach are given in Table 1.

**TABLE 1:**
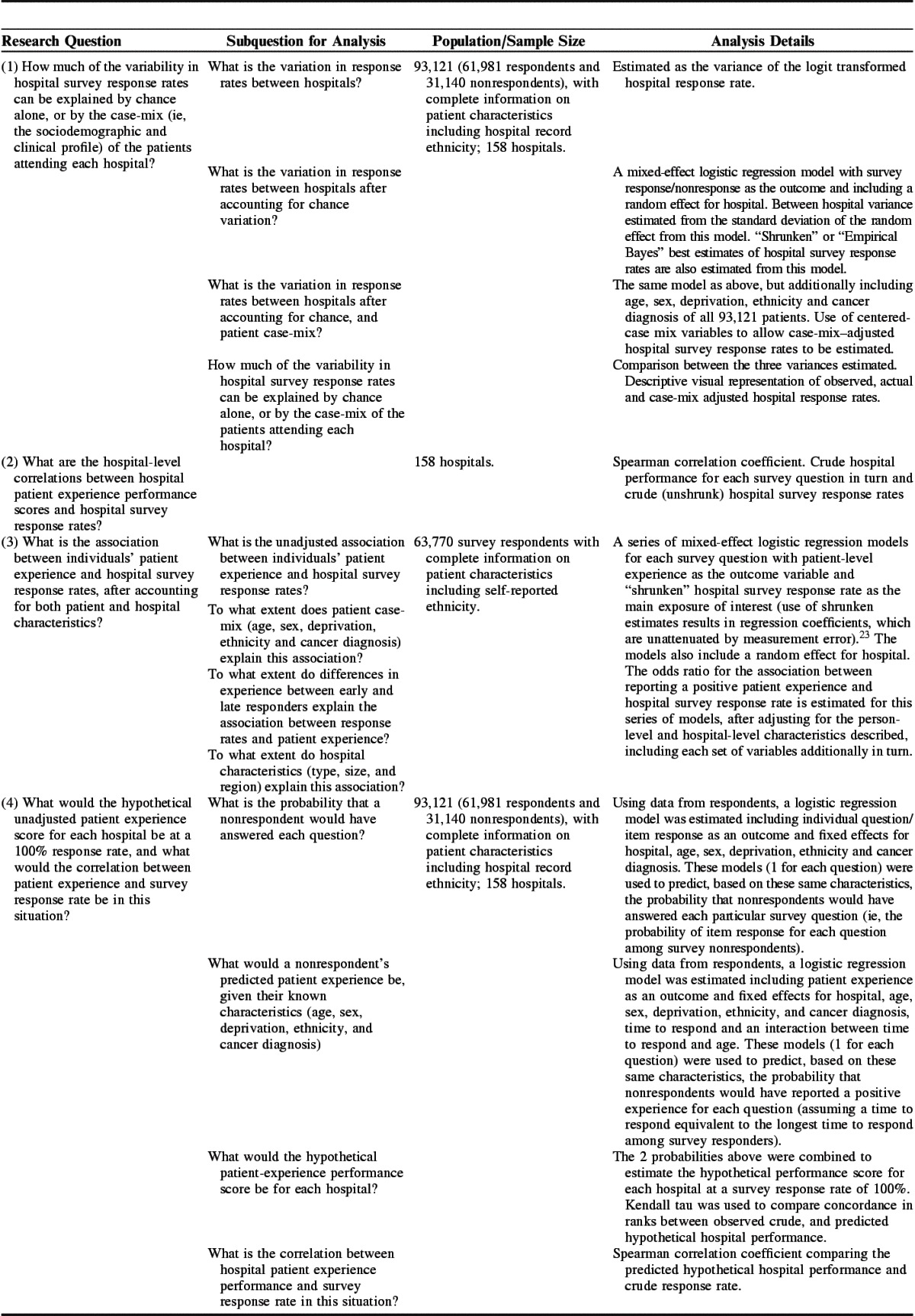
Research Questions and Analysis Details

In sensitivity analyses we explored the findings from analyses predicting the hypothetical experience of nonresponders from models including and excluding the time to respond variable in this prediction.

All analyses were initially carried out for all 60 evaluative survey questions. For clarity, results are presented here for only 9 questions that represent the range of the findings across the 60 items. Those 9 questions were selected on the basis of the third analysis, representing 3 questions each with (a) the strongest associations between hospital response rate and performance, (b) associations at the mid-point, and (c) the weakest (or negative) associations. Full findings are presented in Supplemental Digital Content 3 (http://links.lww.com/MLR/B52), Supplemental Digital Content 4 (http://links.lww.com/MLR/B53), Supplemental Digital Content 5 (http://links.lww.com/MLR/B54), Supplemental Digital Content 6 (http://links.lww.com/MLR/B55). For 1 question (experience of chemotherapy treatment) regression models failed to converge; summary and full findings are therefore presented for 59 questions only.

Analyses using information about nonrespondents from the dataset with the characteristics of all patients sent the survey used hospital record ethnicity coding; however, analyses based on respondents alone used self-reported ethnicity, as this is the gold standard.^24^ Full details of the exact sample of patients included in the 4 analyses are given in Table 1. All analyses were performed using Stata 13.0.^25^

## RESULTS

The overall response rate to the survey was 66.5%, with 67,713 total responses received (full respondent flow chart, Supplementary Digital Content 2, http://links.lww.com/MLR/B56). Hospital response rates ranged from 38.9% to 77.4% (Table 2). Full details of each survey question and national average performance are presented in Supplemental Digital Content 3 (http://links.lww.com/MLR/B52). Low–response rate hospitals were more likely to be teaching hospitals, and in London. Patients from low–response rate hospitals were more likely to be young, from more deprived areas, and to have taken longer to return their surveys (Table 2).

**TABLE 2:**
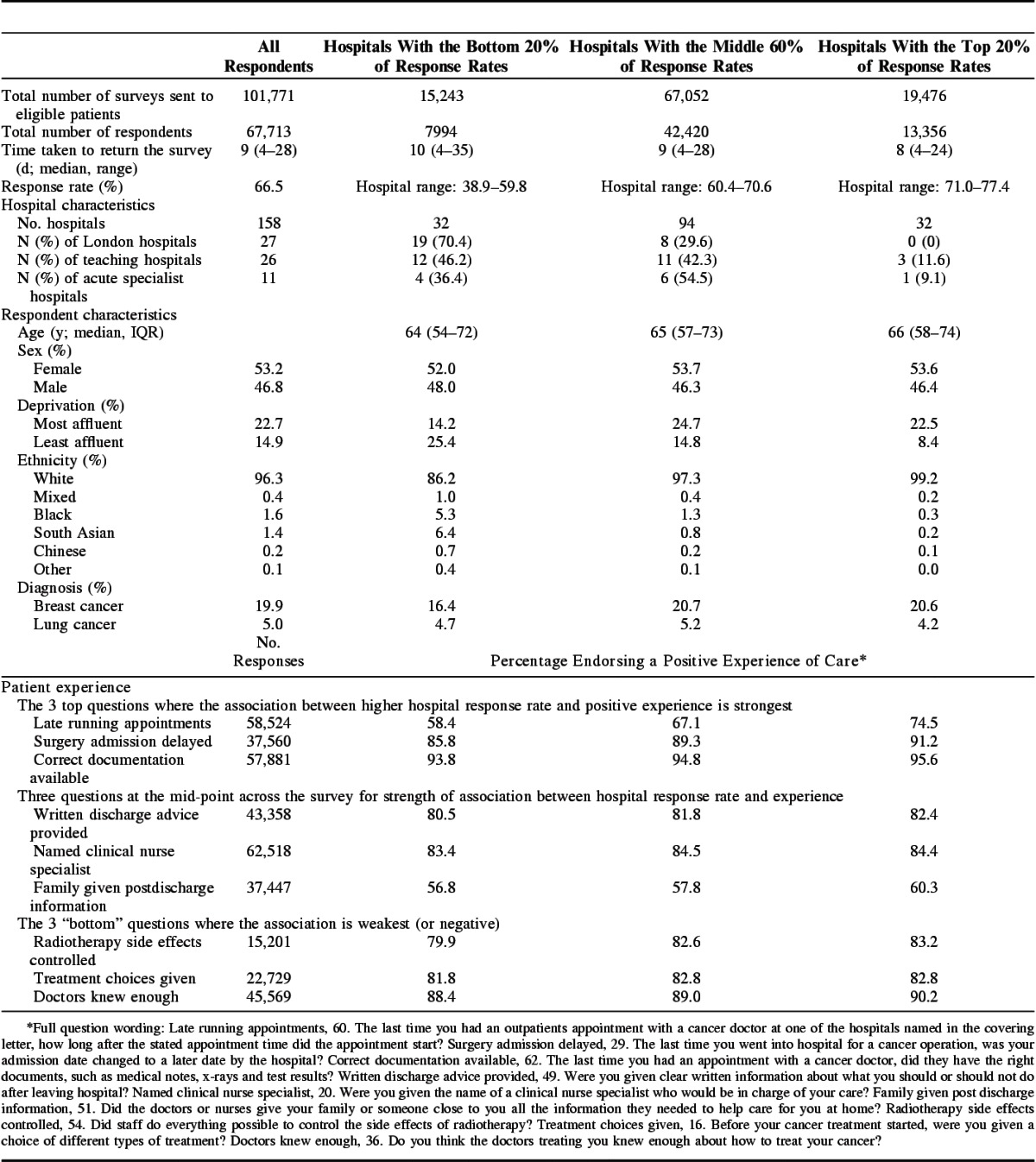
High, Medium, and Low Response Rate Hospitals: Hospital and Patient Characteristics, Patient Experience

**FIGURE 1:**
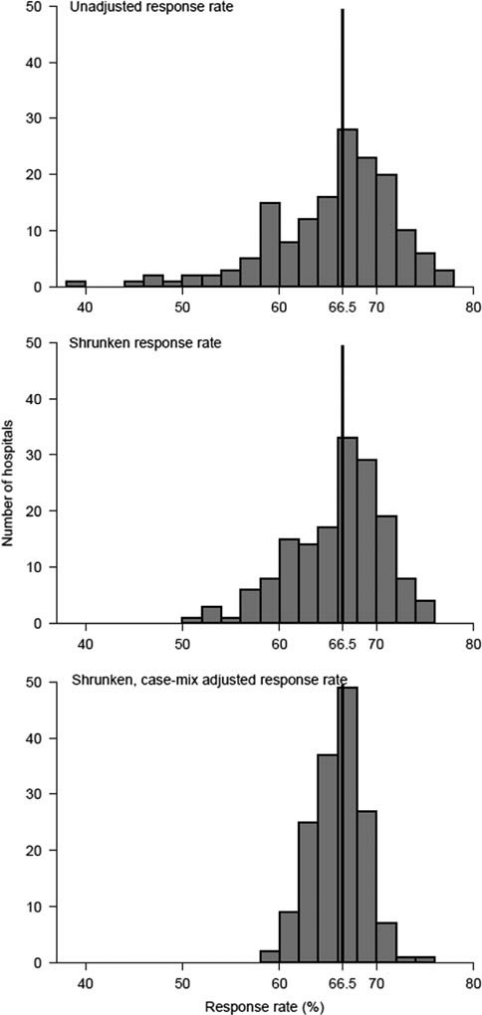
Unadjusted (crude), shrunk (best estimate), and case-mix adjusted hospital survey response rates. Case-mix adjusted survey response rates are estimated assuming all hospitals had the “average” patient case-mix.

In analysis 1 we found that chance explained a small amount (32%) of the variation in hospital response rates (Fig. 1, comparison of top 2 panels). In contrast, patient case-mix differences between hospitals explained a further 58%, with case-mix–adjusted hospital response rates ranging from 58.9% to 75.4% (Fig. 1, bottom panel).

**FIGURE 2:**
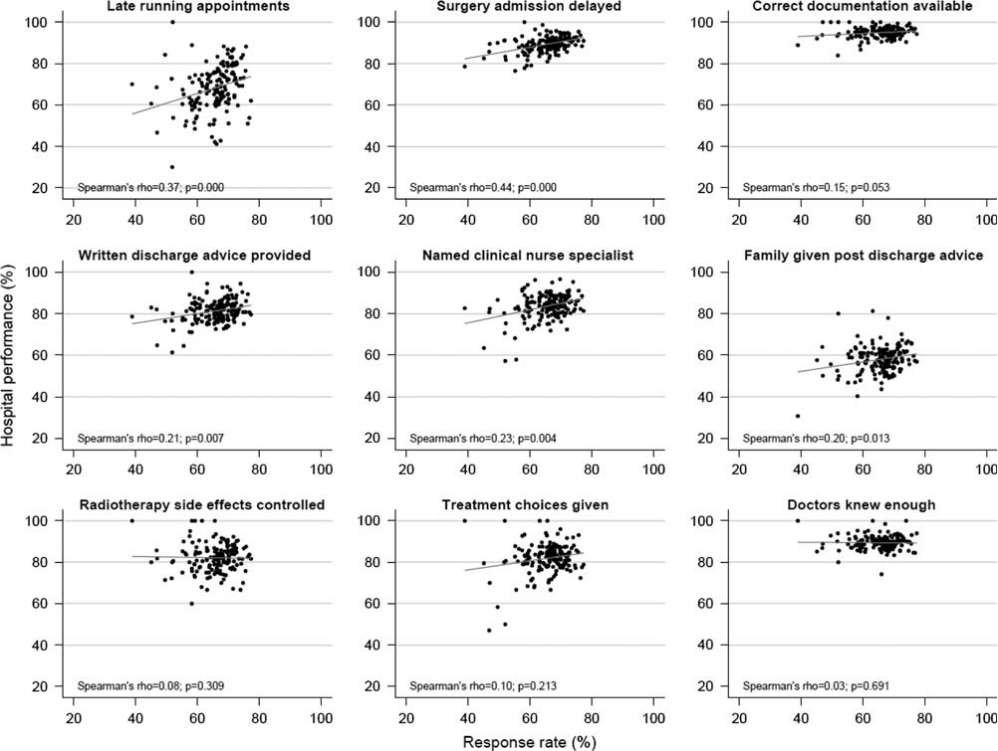
Unadjusted correlation between hospital response rate and hospital performance (% endorsing a positive response at each hospital).

In analysis 2 we found that for all questions there was a positive hospital-level correlation between response rate and hospital performance. The Spearman correlation coefficient varied from 0.03 to 0.44 across questions, reflecting more positive experience scores in high–response rate hospitals (Fig. 2).

The results from the third analysis (logistic regression) are presented in Table 3 (top row). There was evidence (*P*<0.05) that patient experience and response rates were positively associated for 53/59 questions. The estimated odds ratio for reporting a positive patient experience associated with attending a hospital with a 10% higher survey response rate ranged from 1.07 to 1.51 across survey items. Effect sizes typically became attenuated after adjusting for patient case-mix [odds ratio range, 0.94–1.56 (*P*<0.05 for 39/59 questions)], but that adjusting for patient time to survey response makes little additional difference. Associations do attenuate further [range, 1.04–1.30 (*P*<0.05 for 25/59 questions)] when additionally adjusting for hospital characteristics. The reduction in the number of questions with evidence (*P*<0.05) that reporting a positive patient experience was associated with attending a hospital with a higher survey response rate occurred primarily because association strength reduced, rather than because estimates became more imprecise.

**TABLE 3:**
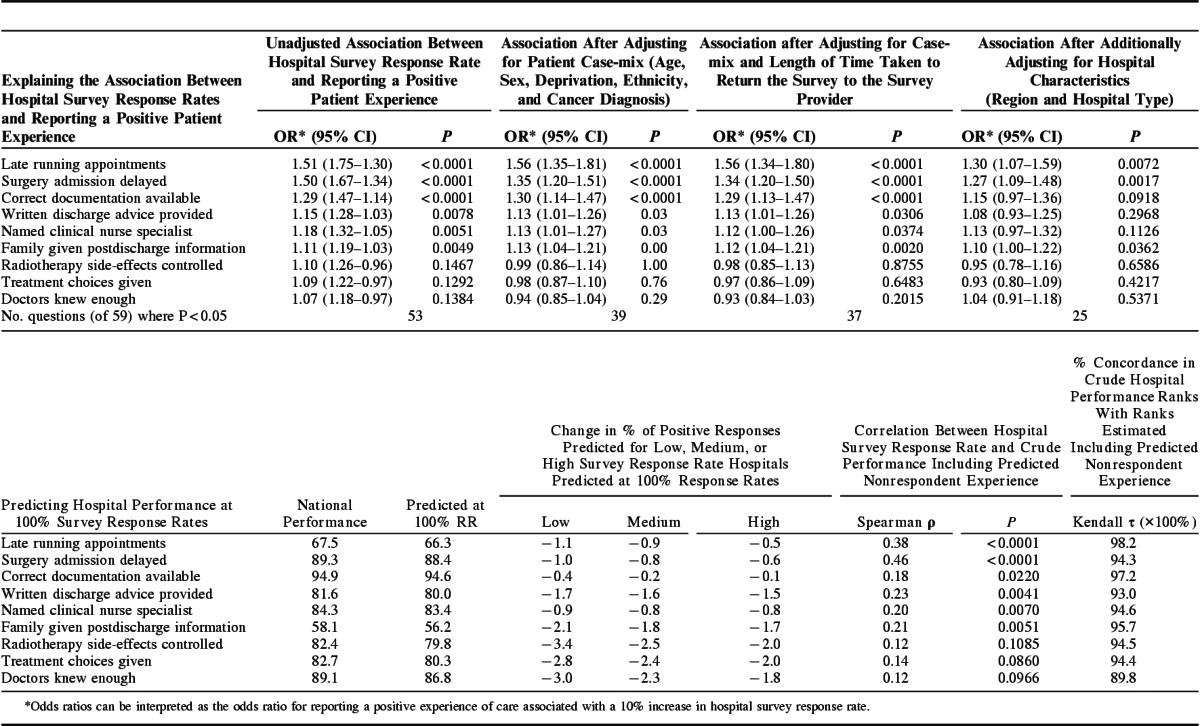
Regression Modeling Findings; the Association Between Hospital Survey Response Rate and Reporting a Positive Patient Experience, Before and After Adjusting for Patient and Hospital Characteristics; and Findings After Including the Predicted Experience of Survey Nonrespondents

Of the 9 exemplar questions, the 3 with the strongest positive association between hospital response rate and patient experience all relate to administrative processes of care. This is in contrast in particular with the 3 questions with weak/negative associations, which all measure direct patient evaluations. We followed up this observation by considering all items across the survey. Of the 15 administrative items in the whole survey, 10 have stronger-than-median associations with the organization response rate [*P*=0.018, Kruskal-Wallis rank test (Supplemental Digital Content 4, http://links.lww.com/MLR/B53)]. We also found that region (particularly, whether a hospital is in London), rather than hospital type, was the more important hospital characteristic explaining the association between patient experience and hospital response rates. Full findings appear in Supplemental Digital Content 4 (http://links.lww.com/MLR/B53).

The results of the fourth analysis appear in Table 3 (bottom row), and demonstrate that after predicting hypothetical hospital performance scores with complete response (a 100% response rate), concordance in hospital performance ranks with the crude unadjusted scores is very high, ranging from 89.8% to 98.2%. On average, the predicted complete-sample national scores are 2.4% lower than the crude; the difference between the 2 scores tends to be larger for low–response rate hospitals. This means that findings from this analysis resulted in a larger variation in hospital scores, compared with respondents alone. Correlation coefficients between hospital response rate and performance become slightly stronger (0.13–0.46) after including predicted estimates from nonrespondents compared with the crude performance. Full findings from these models and all sensitivity analyses are presented in Supplemental Digital Content 5 (http://links.lww.com/MLR/B54)and Supplemental Digital Content 6 (http://links.lww.com/MLR/B55).

## DISCUSSION

In CPES, hospitals with higher response rates tend to have higher experience scores. Although variation in case-mix explains a substantial proportion of variation in response rates between hospitals, there remains a positive association between hospital survey response rate and patient experience, even after adjusting for patient case-mix and time taken to respond to the survey.

The use of patient-level predicted responses assuming a hypothetical 100% response rate (a) has concordance in performance ranks compared with crude scores, which are very high, (b) reduces national average scores overall, (c) reduces the scores of low–response rate hospitals more so than it does for high–response rate hospitals, and so (d) strengthens rather than reduces positive associations between hospital performance and hospital response rate.

These observations suggest that, although there appears to be nonresponse bias present, it is unlikely that the association between hospital scores and response rate are driven by this bias. Rather, given that the positive association appears to be strongest for questions relating to administrative processes, and that a substantial proportion of the association is explained by hospital characteristics, it may be that 1 or more hospital-level factors are driving both hospital score and response rate (including the quality of care provided). Importantly, any nonresponse bias that is present is in the opposite direction to the usual concerns and, if anything, underestimates the disparities between hospitals.

There are plausible reasons why this might be the case. For example, hospitals that emphasize patient experience may actively encourage all patients to return the survey. Alternatively hospitals with better administrative processes may both provide better patient experience and more accurately maintain patient contact information, facilitating response to surveys. Survey response rates may be an endogenous marker of quality of care, rather than a reflection of individual nonresponse bias.

### Findings in the Context of Previous Work

Often, a prime motivation for the pursuit of high response rates is to minimize the potential for nonresponse bias. However, CPES has a high response rate (66.5%) and yet displays other signs often taken as indicators of bias, that is, differential response rates between hospitals and patient groups, and an association between response rate and performance.

Nonresponse can have at least 6 different drivers: (1) chance, (2) observed patient case-mix, (3) unobserved patient mix, (4) a direct relationship with the survey outcome (patient experience either at a patient or (5) organization level), or (6) ecological (organizational) sources.^9^,^10^

We find that both chance and patient case-mix are associated with the variation in response rates seen between hospitals, but we do not find that they substantially explain the association between patient experience and hospital survey response rate. This is consistent with previous work, which found that variation in response by different patient groups is not a reliable indicator of inherent nonresponse bias.^26^ Case-mix adjustment reduces bias in comparisons between organizations due to variation in patient characteristics,^12^,^27^,^28^ and importantly, can also improve the perceived fairness of these measures, improving clinician and manager engagement in improvement efforts.^29^ Specifically, regarding nonresponse, where the same patient characteristics are associated with both patient experience and with survey response^14^,^30^–^33^ then case-mix adjustment will account for these differences and will allow fair comparisons.

Unobserved case-mix is a third possible driver and is a concern for any study. However, previous findings indicate that overall case-mix adjustment of hospital scores makes only a small difference for this survey. Together with the fact that case-mix differences between NHS hospitals in England are relatively small, argues against unobserved case-mix being the primary driver.^11^

Fourth, a direct patient-level relationship between patient experience and survey response in which people who receive poorer care are less likely to respond to requests to report their experiences.^34^ We found that adjusting for survey response time, as a proxy measure for this explained a very small amount of the association between patient experience and hospital-level response rates. This is consistent with previous work.^8^ It may be appropriate to adjust for survey response time, but this patient-level relationship does not explain the association between patient experience and hospital-level response rates.

Finally, previous epidemiological work has found that both area-level and individual-level factors are important in nonresponse^35^,^36^ and the observation that group-level factors are important drivers of survey nonresponse may well be relevant here. We find that adjusting for one particular characteristic of hospitals (being a London hospital) is best at explaining the association between hospital survey response rate and patient experience. Findings that associations between response rates and patient experiences are strongest for items relating to administrative processes suggest that hospital rather than patient-level characteristics are driving at least some of the observed correlations. Previous work for this survey found a very large variation in the amount of missing data in routine data collection (for ethnicity) between hospitals^24^ and this would provide further indirect evidence for variation in administrative quality (therefore, potentially, address recording) between hospitals.

Our finding that predicting the experience of nonrespondents based on the case-mix and predicted experience of nonrespondents decreased the overall estimated mean national experience but strengthened the association between hospital-level response rate and patient experience is consistent with predictions.^13^

### Strengths and Limitations

A major strength of this analysis is the high but variable response rate for this survey. This allows us to present some evidence that may help to disentangle the issues of low response rates and differential response rates between organizations.

There are 4 limitations to this work worth highlighting.

First, the issue of unmeasured case-mix is discussed above and is unlikely to be a major concern in this setting, although it should be noted that organization-level characteristics may stand in for unobserved individual-level characteristics that differ between organizations.

Second, in line with best practice^21^ we excluded a small number of eligible patients where surveys were returned to the survey provider because of having moved house, but for nonrespondents the accuracy of recorded addresses is simply not known. Our suggestion that variation in response rates may reflect variation in the accuracy of recorded patient addresses assumes that for most respondents where addresses are incorrectly recorded this is not known; the known exclusions would tend to attenuate the magnitude of this effect.

Third, there are limitations to using survey response time as a predictor of the (usually poorer) experience of nonrespondents^37^; for example, these patients may simply remember their experiences of care less clearly; predictions based on the case-mix of nonrespondents alone, however, gave a consistent direction of effect.

Finally, the analysis presented here assumes that the mean experience that nonresponders would have reported had they responded can be predicted from only those variables included in the models (including the time-to-respond variable). If, however, patient experiences affect propensity to respond beyond this, the only way to assess this impact is to aim to elicit reported experience from nonresponders. However, the efforts required to do this may well affect the reported experience or other aspects of the data quality directly, potentially making such approaches ineffectual.^38^,^39^

### Recommendations for Policy and Practice

First, it is important for people using patient experience surveys to recognize that findings may overestimate true national mean experience to some extent, especially when response rates are low. Second, patient-level case-mix adjustment, possibly including an adjustment for response time, will improve fairness, and perceived fairness of comparisons, and in the context of differential nonresponse between patient groups case-mix adjustment will account for this in organization comparisons. In the United States, case-mix adjustments are routinely applied to patient experience measures used for organization comparisons; however, in the United Kingdom findings from this survey are reported without adjustment, and for national surveys in primary care and acute hospitals, findings are weighted to the organization population.

However, adjusting for hospital-level characteristics or survey response rates is not recommended, even where a correlation between hospital-level survey response rates and patient experience is observed. Patient experience is known to be poorer in London, for example,^40^,^41^ but adjusting for this hospital-level characteristic when making comparisons between organizations would adjust away true variation in the quality of care provided.^12^ We cautiously posit that the association between patient experience and organization survey response rates may be driven by administrative or other factors relating to care quality, and again it would not be appropriate to adjust for this before making performance comparisons between organizations.

We find that survey response rate alone is a poor indicator of bias, and reiterate that it should not be used as a stand-alone measure of the validity of survey findings. We recommend best practice in maximizing response rates^42^ and survey-specific evaluations,^5^–^7^,^36^ rather than response rates, be used to assess bias. The possibility that variation in response rates is an indirect indicator of care quality should not be ignored. Improving performance and the quality of care provided by low-performing organizations could be expected to increase response rates accordingly.

## CONCLUSIONS

There are persistent concerns about associations between patient experience and hospital-level survey response rates. We find that the case-mix of respondents, known characteristics of survey nonrespondents, and the person-level relationship between nonresponse and patient experience do not fully explain this association. This should reassure stakeholders using survey findings to improve care quality. Case-mix adjustment of patient characteristics, possibly also including an adjustment for response time, can improve the fairness of organization comparisons. Low or high response rates alone are not an indicator that findings from a particular hospital are more or less likely to be biased and adjustment of hospital patient experience performance scores for hospital-level characteristics or response rates is not recommended.

## Supplementary Material

SUPPLEMENTARY MATERIAL
