# Supplementary material for: Do Differential Response Rates to Patient Surveys Between Organizations Lead to Unfair Performance Comparisons? Evidence From the English Cancer Patient Experience Survey
Source: Med Care. 2015 Dec 18;54(1):45–54. doi: 10.1097/MLR.0000000000000457 (PMC4674144; doi:10.1097/MLR.0000000000000457)
Supplement: SUPPLEMENTARY MATERIAL [file mlr-54-45-s001.docx]

**Parameterising the relationship between the time to respond to the survey and patient experience**

Based on the survey provider date system for logging the survey responses, a measure of the length of time taken to return the survey was available for respondents, allowing analyses based on the assumption that late respondents are more similar to nonrespondents than early respondents.

This approach, or an approach based on wave analysis (i.e. whether respondents returned the survey after on, or more than one reminder) is a method that has been proposed to understand the potential implications of nonresponse in survey research.^1 2^ Previous work has also used time to respond measured as a rank-based metric, within hospital, for case-mix adjustment for hospital patient experience performance scores^3^ (although it is worth noting that in that particular context different hospitals used different survey modes (i.e. postal survey, phone survey, or mixed mode).

In this paper (Do differential response rates to patient surveys between organizations lead to unfair performance comparisons? Evidence from the English Cancer Patient Experience Survey) the time to respond to the survey is used in two ways:

- We adjust for time to respond in the analysis where we explore the relationship between reporting a positive patient experience and hospital survey response rate. Our work found that the differences in patient experience between early and late survey responders were not important in explaining the relationship between hospital-level survey response rates and patient experience
- We also use this variable as part of the model where we predict the patient experience of nonresponders in each hospital. In this analysis we assume that all nonresponders have the same experiences as the people who responded most slowly to the survey (i.e. the experience of people who took about 18 weeks to return the survey to the survey provider). In our main and sensitivity analyses we found that these predictions are consistent with predictions based on case-mix alone.

There are, however, limitations to using survey response time as a predictor of the (usually poorer) experiences of survey nonrespondents.^4^ In this appendix we describe some of our initial work exploring this measure, and some of the assumptions that are underlying the analyses that we present. We explore three main questions.

1. What is the distribution of the time taken to return the survey?
2. Whether a linear, quadratic, or other relationship is the best way to model the relationship between being an early or later responder and patient experience?
3. Whether there was any evidence that the relationship between time to respond and reporting a positive patient experience varied between different groups of patients, by age, gender, ethnicity, deprivation or cancer diagnosis?

**Time taken to respond to the survey**

In order that postage time is not included we have a working definition of time taken to respond to the survey which is the difference between the date each response was logged as received and the date on which the first response was logged. The distribution of “time taken to return the survey” across all respondents is presented in figure 1a, with a histogram presenting the number of responses logged per day, with the superimposed line representing the cumulative survey response rate at each time point. A 40% response rate is achieved within about 4 weeks of the first response being returned; We can see that responses are not logged by the survey provider on Saturdays or Sundays, and that there is a corresponding slight increase in numbers of responses logged on Mondays. There is peak in responses received between 1 and 2 weeks, a second (smaller) peak between 3 and 4 weeks, and a third (smaller again) between 6 and 7 weeks. It is likely that these peaks correspond to the first a second reminders sent out. All responses included in this analysis were received within 19 weeks of receipt of the first response by the survey provider.

**The relationship between time taken to respond to the survey and reporting poorer patient experience**

In our preliminary analyses of this variable we explored whether assuming a linear relationship was appropriate for the relationship between time taken to respond to the survey and reporting positive patient experiences. We found that across almost all questions later respondents are less likely to report positive experiences than early respondents. We also noted that the odds ratios from models that include a squared term were broadly consistent with those that did not. Often the squared term was statistically significant, but the deviation from linearity was small. For some questions the linear model alone slightly over-estimated the strength of relationship between returning a survey at later time points. For consistency, we chose the simpler (linear) model parameterisation and as an extreme case-sensitivity analysis we compared our predictions of patient experience among nonrespondents with and without time to respond, in order to describe the maximum level of possible over-estimation that may have occurred, and found that predictions were consistent using both approaches, .

Previous work has also used time to respond measured as a rank-based metric within hospital for case-mix adjustment.^3^ As we noted above in that particular context different hospitals used different survey modes and so a method which accounted for differences in response speed dependant on mode was deemed necessary. In the context of CPES the same mode (postal) is used for all hospitals. In initial modelling work on this project we did explore this approach and found that it improved the fit of the model compared with a model with a linear parameterisation for some questions, but we did not explore this further for two reasons, first, the coefficients from a linear model are more directly interpretable, and we were also interested in using an individual, rather than hospital based measure (as this is included as a separate variable) for predicting the experience of nonresponders.

**Does the relationship between time to respond and reporting a positive patient experience vary between different groups of patients?**

Finally we explored whether there was any evidence that the relationship between time to respond and reporting a positive patient experience varied between different groups of patients, by age, gender, ethnicity, deprivation or cancer diagnosis. We ran six series of models, including each interaction separately, and considering age as both a continuous and categorical variable. We considered all 59 survey questions, and recorded the interaction p-value from each. We plotted the p-values in a p-p plot to explore whether the p-value distribution varies from the uniform distribution that would be expected by chance alone. Findings are presented in Figure 1b. In the situation where no interactions existed we would expect that the points on the p-p plot would lie on the diagonal line (i.e. that 5% of models have p<0.05, 10% of models have p<0.1 etc.) this is predominantly the case for most variables. The exception is for the series of models including interactions between time to respond and patient age (either as a categorical or continuous) where substantial deviations from the diagonal are seen implying the presence of interactions not due to chance. The implication of this is that the relationship between time to respond to the survey and reporting a more positive patient experience does vary by age (although not by the other variable) and there was stronger evidence for an interaction using the age categories as a continuous variable, and so we included this interaction in our prediction model.

**References**

1. Johnson TP, Wislar JS. Response rates and nonresponse errors in surveys. JAMA : the journal of the American Medical Association 2012;**307**(17):1805-6.

2. Halbesleben JRB, Whitman MV. Evaluating Survey Quality in Health Services Research: A Decision Framework for Assessing Nonresponse Bias. Health Serv Res 2013;**48**(3):913-30.

3. Elliott MN, Zaslavsky AM, Goldstein E, et al. Effects of survey mode, patient mix, and nonresponse on CAHPS hospital survey scores. Health Serv Res 2009;**44**(2 Pt 1):501-18.

4. Olson K, Groves RM. An Examination of Within-Person Variation in Response Propensity over the Data Collection Field Period. J Off Stat 2012;**28**(1):29-51.

**Figure 1a, the distribution of time taken to return the survey from the date that the first response was logged. A 40% response rate is achieved within about 4 weeks**

**Figure 1b. Interaction p-values**
