# Supplementary material for: Do Differential Response Rates to Patient Surveys Between Organizations Lead to Unfair Performance Comparisons? Evidence From the English Cancer Patient Experience Survey
Source: Med Care. 2015 Dec 18;54(1):45–54. doi: 10.1097/MLR.0000000000000457 (PMC4674144; doi:10.1097/MLR.0000000000000457)
Supplement: SUPPLEMENTARY MATERIAL [file mlr-54-45-s002.docx]

**Supplemental digital content 2: Participant flow chart and sample derivation**


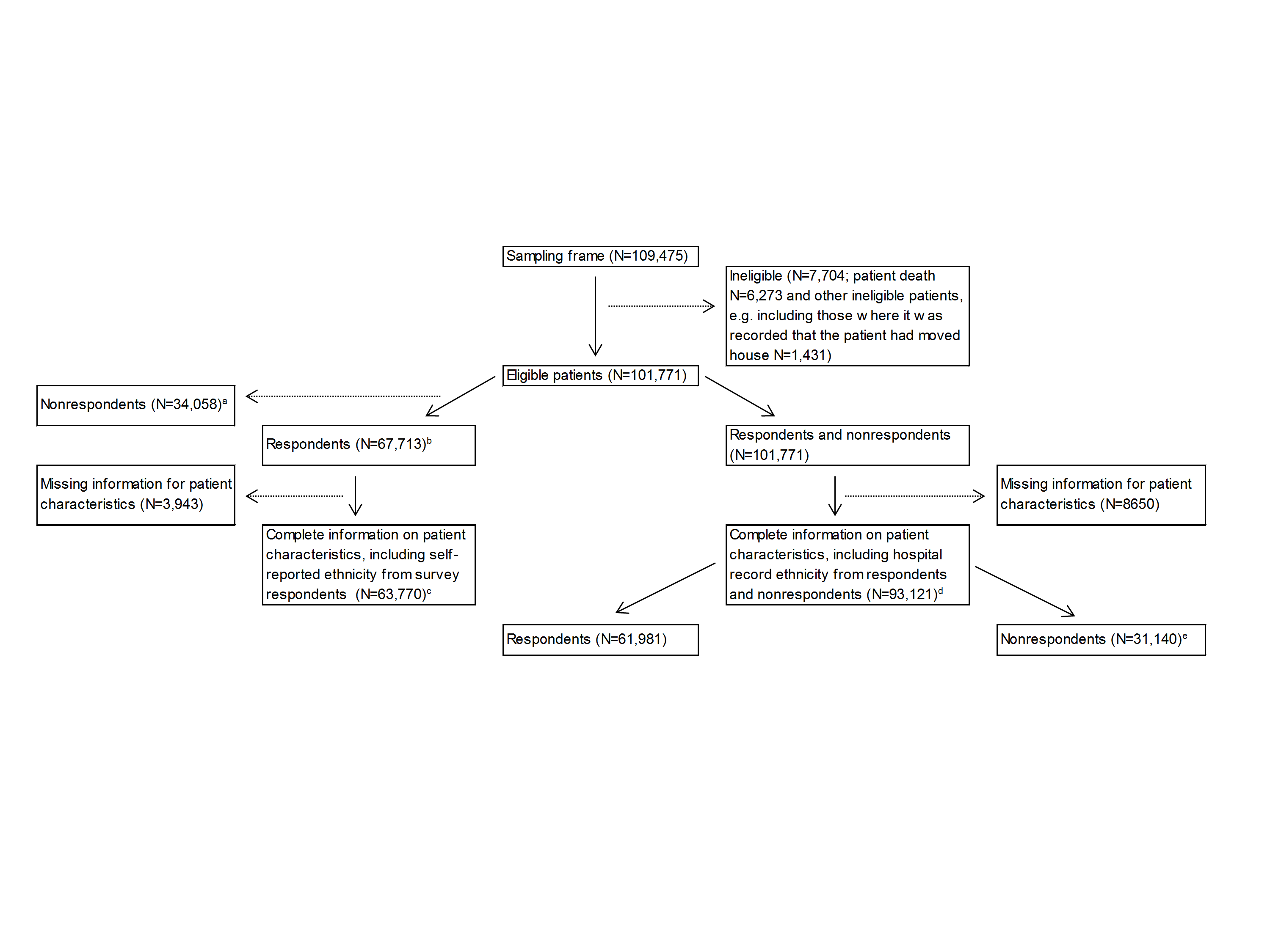


Regarding analyses: Overall and hospital specific survey response rates were calculated for all nonrespondents (a) and respondents (b). Logistic regression analyses looking at how patient and organization characteristics explain the association between hospital response rate and patient experience were carried out for survey respondents with complete patient characteristics information in analysis 3 (c). - Case-mix adjusted hospital response rates – analysis 1 - were estimated from a model including all patients in the sampling frame with complete patient characteristics information (d). In analysis 4 predicted patient experience among nonrespondents were estimated from the same population (d) and experience was predicted for nonrespondents with complete patient characteristics (e). This was compared with the patient experience of all respondents (b).
